# Visual and motor skills development in preterm and full-term infants: A protocol for longitudinal data collection

**DOI:** 10.1016/j.mex.2025.103546

**Published:** 2025-07-30

**Authors:** Ana Isabel Ferreira, Alice Nunes, Cláudia Silva, Teresa Tomé, Cláudia Quaresma, Carla Quintão

**Affiliations:** a Laboratory of Instrumentation, Biomedical Engineering and Radiation Physics, Physics Department, Nova School of Science & Technology – NOVA University of Lisbon, Portugal; b Associated Laboratory in Translation and Innovation towards Global Health (REAL), NOVA School of Science & Technology, NOVA University of Lisbon, Portugal; c Higher Health School, Polytechnic Institute of Beja, Portugal; d São José Local Health Unit, Lisbon, Portugal; e Higher School of Health Technology of Lisbon, Polytechnic Institute of Lisbon, Portugal; f Alcoitão Higher School of Health, Santa Casa da Misericordia of Lisbon, Portugal; g Lusíadas Hospital, Lisbon, Portugal

**Keywords:** Extremely preterm, Very preterm, Full-term, Electrophysiological Assessment, Visual Evoked Potentials, Electrodermal Activity, Development, Evaluation

## Abstract

Preterm birth, defined as birth before 37 weeks of gestation, affects approximately one in ten live-born infants globally. The risks and potential complications are more pronounced in infants born before 32 weeks, particularly those born before 28 weeks, necessitating specialized monitoring of developmental outcomes. Traditional developmental assessments often rely on performance-based observations, which can be subjective, and may not detect subtle or delayed difficulties that emerge later, such as in school-age children.

This study presents the development and pilot application of a comprehensive protocol designed for the objective and integrated collection of clinical, developmental, and electrophysiological data to evaluate the impact of preterm birth. The protocol involves monitoring two groups of healthy infants—preterm and full-term—at four time points during the first year (4, 6, 9, and 12 months). Assessments include clinical evaluations, the Bayley Scales of Infant and Toddler Development, and electrophysiological recordings such as visual evoked potentials (VEP), electrodermal activity (EDA), and an ophthalmological screening assessment at 4 months.

Two case examples (one full-term, one preterm) are provided to illustrate data collection and application. While the article does not include outcome analysis, it emphasizes protocol structure and applicability, offering a foundation for future longitudinal study.

## Graphical abstract

**Figure:**
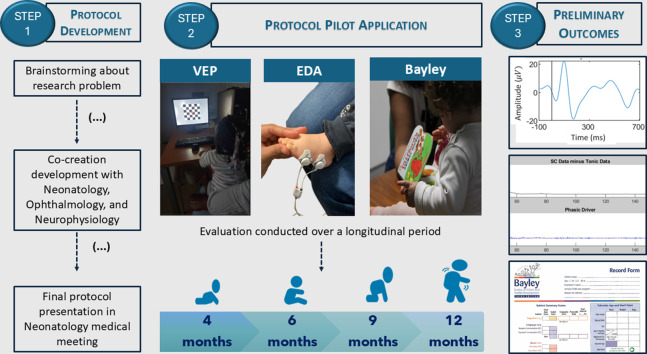

## Specifications table

**Table:** 

**Subject area**	Neuroscience
**More specific subject area**	Preterm infant development monitoring
**Name of your protocol**	Visual and motor development in preterm infants (VMD-PTI)
**Reagents/tools**	• Clinical and socio-demographic variables; • Bayley Scales of Infant and Toddler Development; • Participation and Activity Inventory for Children and Youth (PAI-CY); • Teller Cards application based on preferential looking method; • Visual performance assessment through observation; • Gnautilus from GTEC; • Biosignalplux from PLUX.
**Experimental design**	Protocol was developed using a co-creation methodology, and a workflow composed of seven steps was used. This protocol will be applied in a cohort study that follows two groups of infants longitudinally (preterm with gestational age ≤ 32 weeks and full-term infants with gestational age ≥ 38 weeks) during the first year of life.The infants are assessed in four key moments for development follow up (4, 6, 9 and 12 months). The protocol included: • The monitoring of clinical data variables (eg: spontaneous or medical assisted pregnancy, gestational age, APGAR index), • The assessment of global development at 4 and 12 months of age; • The monitoring of infants’ participation and activity involvement at 4 and 12 months of age; • The acuity and ocular motricity screening assessment at 4 months; • The quantification of visual evoked potentials (using a checkerboard pattern) at 4, 6, 9 and 12 months of age; • The quantification of electrodermal activity at 4, 6, 9 and 12 months of age.
**Trial registration**	Not applicable
**Ethics**	The research project was approved by ethical commissions of both health institutions involved in protocol development and data collection (Ethical Commission of São José Local Health Unit and Ethical Commission of Baixa Family Health Unit). Both health institutions gave the ethical approval with the references INV 14 and 6156/CES/2020, respectively.
**Value of the Protocol**	• Protocol results from a co-creation approach developed between researchers and clinical professionals; • Assessment protocol that integrates data from clinical variables with traditional tools (such as Bayley Scale of Infant and Toddler Development) and electrophysiological data (VEP and EDA); • Longitudinal protocol that included 4 assessment points during the first years of life; • Protocol included two groups of healthy infants (preterm with gestational age ≤32 weeks and full-term with ≥ 38 weeks gestational age).

## Background

Preterm birth affects 15 million births per year worldwide and refers to infants born alive before 37 weeks of pregnancy are completed [1], with possible impact during the life course [2,3]. Preterm infants can be grouped according to gestational age as follows: moderate to late preterm (32 to 37 weeks), very preterm (28 to 32 weeks), and extremely preterm (<28 weeks) [1]. The risk of developmental sequelae increases with decreasing gestational age, low birth weight, and/or the presence of major neurological lesions [1,4]

Preventive measures, ongoing monitoring, and, when necessary, treatment of sequelae demand concerted efforts from clinical and research teams as well as international organizations [5,6]. Taken together, these challenges highlight the need for an integrated, longitudinal, and multimodal protocol capable of identifying early indicators of developmental risk in preterm infants.

### Preterm infants’ assessment and follow-up

Guidelines for a correct follow-up of preterm infants often rely on specific clinical parameters, including neurological, auditory, and vision screening, as well as developmental tracking [7,8]. In preterm infants, visual skills are usually compromised, from a long-term perspective [9,10] including in situations without retinopathy of prematurity (ROP) [11,12]. Those skills could have diverse influences on motor and cognitive development [13,14].

Related to ophthalmological evaluation, assessing ROP sequelae is fundamental [15], as well as is the acuity and ocular motricity [16]. Concerning visual function, parental questionnaires focusing on the impact of visual dysfunction on daily life can be helpful [17].

To gain a deeper understanding of the function and integrity of the visual system, electrophysiological assessments can be employed. Visual electrophysiological signals afford direct, quantitative, and objective assessment of visual pathway function at different levels and thus complement imaging or psychophysical tests [18]. Different studies argue that pattern reverse stimuli is the preferred stimuli in clinical situations because of its low variability in waveform and timing [19,20]. The electrophysiological pattern develops during the first years of life, and the parameters most assessed include latency and amplitude of complex N75 / P100 / N135 [21,22].

EDA refers to dynamic fluctuations in the skin’s electrical properties, primarily modulated by sudomotor activity under the control of the sympathetic branch of the autonomic nervous system [[23], [24], [25]]. EDA measurements in infants are less common compared to other age groups and, when conducted, are typically associated with pain stimuli [[26], [27], [28]].

The EDA fluctuations are influenced by several intrinsic skin properties, including epidermal thickness, hydration levels, and sweat gland distribution.

In preterm infants, the immaturity of skin development has been well documented and is associated with increased transepidermal water loss and altered skin barrier function [29,30]. Simultaneously, autonomic nervous system (ANS) regulation in this population appears to be underdeveloped, with empirical evidence indicating dysregulation of sympathetic activity [[31], [32], [33]]. Additionally, according to the model of synactive organization of behavioral development, ANS interacts with the motor and attentional/interactive systems in preterm infants [31]. More recently, the influence of the ANS on motor development during fetal life as well as in a long-term perspective has also been explored [34].

### Preterm birth impact outcome: what needs to be answered?

The surveillance of preterm births is a routine practice, with particular emphasis on evaluating and monitoring developmental sequelae. However, the use of longitudinal approaches during the early months of life, combined with integrated methodologies that incorporate clinical, developmental, and electrophysiological data, remains uncommon in both clinical and research contexts. This dearth is primarily attributable to the challenges inherent in such methodologies, including resource-intensive demands and participant compliance constraints.

Within this context, the current study aims to enrich the understanding of the developmental impact of preterm birth, more specifically the infants with 32 (or less) weeks gestational age, but without major neurological damages. By employing a longitudinal framework and integrated data analysis techniques, this investigation endeavours to bridge existing gaps in knowledge. It is also important to investigate which variables have the greatest influence on infants' visual and motor development, and whether it is possible to identify a specific age within the first year of life that is more accurate for predicting a poor prognosis.

### Methodology used for protocol development

**Fig. 1:**
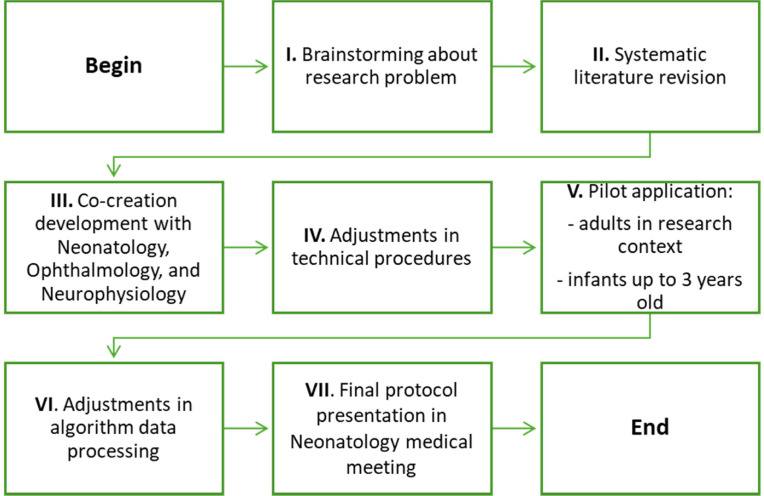
Methodology for protocol development.

The protocol was developed through a co-creation approach in collaboration with the Neonatology, Ophthalmology, and Neurophysiology teams at the São José Local Health Unit. The development process spanned approximately eight months and followed a structured sequence of seven phases, as illustrated in Fig. 1.

The project idea originated from the therapeutic experience of the first author in the developmental follow-up of preterm infants. Building on this, a preliminary literature review focused on visual skills assessment and the use of technology was conducted during Stage II [35,36]. The literature review highlighted the importance of incorporating technology into infant assessments to enable more objective and systematic monitoring of early development [36]. Additionally, a limited number of tools were identified for assessing visual performance in children under two years of age and none of them translated to Portuguese. As a result, the Participation Activity Inventory – Children and Youth (PAI-CY) was translated and culturally adapted to European Portuguese [37] within the scope of this research.

During Stage III, structured meetings were conducted with the Neonatology leadership, Ophthalmology team, and Neurophysiology technicians to ensure alignment between the project’s preliminary framework and the technical and clinical requirements. At this phase, several protocol modifications were implemented based on expert clinical input. Notably, the age for the initial assessment, originally scheduled at three months, was revised to four months. This adjustment was informed by developmental considerations highlighted by the clinical teams and was aligned with Stage III deliberations. The updated timeline corresponds more closely with key neurodevelopmental milestones and is consistent with the routine pediatric follow-up schedule recommended by the National Health Authority [38]. This strategic alignment is intended to minimize the burden of additional clinical visits, thereby enhancing the feasibility of protocol integration within standard healthcare pathways for infants and their families, while also promoting participant adherence.

Additionally, the placement of active electrodes was optimized, and a cap was introduced to ensure more accurate positioning—an enhancement developed in collaboration with the Neurophysiology team. The decision to use a single trial for VEP assessment was based on the limited cooperation typically observed in infants and was established during stages III and IV of protocol development, also in coordination with the Neurophysiology team. The type of ophthalmological assessment and the age at which it would be conducted were likewise defined at this stage, in consultation with ophthalmology specialists.

In the subsequent stage, adjustments to technical procedures were made, such as the adjustment in the EDA processing method, enabling continuous data analysis according to the methodology recommended by Benedeck [39]. In Stage V, preliminary applications involving both adults and infants was conducted and some improvements, namely, the introduction of a final Butterworth low pass filter, was done.

Finally, in stage VII of protocol development, presenting it at a Neonatology Medical Meeting facilitated the engagement of all Neonatologists and helped disseminate the research project to families of preterm infants who met the inclusion criteria. Following approval by the Neonatology Medical Team and the Ethics Committees of both health institutions involved, the assessment protocol is considered methodologically structured and ethically approved for pilot application, within the scope of the intended research project.

### Methodology for protocol pilot application

Due to the length of the protocol, the following information is structured into three main subsections:

• Research description;
• Assessment protocol presentation;
• Outcome measure description and contribution to preterm follow up.

Research description
The protocol will be applied in a cohort study that includes two groups of healthy infants— full-term and preterm. The sample will be followed during the first year of life at four specific time points: 4, 6, 9, and 12 months. The preterm group includes extremely preterm infants (gestational age ≤28 weeks) and very preterm infants (gestational age between 28 and 32 weeks), without major neurological lesions.

To achieve the purpose of the study, three main goals have been defined:

• Monitor the global development and biosignals (VEP and EDA) in a full-term and in a preterm born infants in four moments throughout the first year (corrected age in preterm infants);
• Compare the evolution of the two groups of infants under study throughout the first year (corrected age in preterm infants);
• Explore potential correlations between clinical (eg: APGAR index, gestational age, birth weight, type of pregnancy) and demographic variables (eg: has siblings, frequent daycare, mother age) and visual and motor development in preterm infants.

Data collection is being carried out in public healthcare institutions affiliated with the São José Local Health Unit (ULS São José), namely the Dr. Alfredo da Costa Maternity Hospital (MAC), Dona Estefânia Hospital, and the Baixa Family Health Unit, all located in Lisbon, Portugal. Preterm infants are recruited at MAC, while full-term infants are enrolled through the Baixa Family Health Unit.

The recruitment of preterm and full-term infants in distinct healthcare settings reflects the organizational structure of the Portuguese National Health System. Specifically, the preterm infants group, those born at or before 32 weeks of gestational age, are routinely monitored in specialized referral centers, such as MAC. In contrast, healthy full-term infants are followed within primary care settings, such as family health units. This context-driven recruitment strategy ensures alignment with existing clinical care pathways and facilitates the integration of research procedures within routine pediatric follow-up.

The inclusion criteria are:

• born with ≤ 32 weeks gestational age (study group) or born with ≥38 weeks gestational age (control group);
• be followed up in appointments at the MAC (in the case of infants from the study group) or in Baixa Family Health Unit (in the case of the infants from control group);
• be 4 months old at the time of the initial assessment (corrected age in the case of preterm infants);
• parents with fluency in Portuguese or English language.

The exclusion criteria are:

• have major neurological lesions (as grade III periventricular haemorrhage and / or leukomalacia);
• have any developmental disorder;
• have congenital anomalies;
• have congenital infections;
• have chromosomal abnormalities;
• have ROP with plus disease;
• present any other clinical condition or disorder that impacts the typical development;
• do not plan to do the full follow-up program in those health institutions.

Both preterm and full-term groups could include singletons and twins, whether resulting from spontaneous or medically assisted pregnancies. Additionally, infants from both spontaneous and induced births are included. These variables are all systematically collected and incorporated into the data analysis, under the scope of the clinical variables. It is anticipated that the data will be analyzed to determine any potential correlation with subsequent developmental outcomes, as previously described in aims.

Prospective participants for the study group are identified proactively by neonatologists and physicians with expertise in the infants' clinical backgrounds and adherence to research inclusion criteria. At the 2-month corrected age appointment, the study details are presented by the article first author, to the parents, along with a written synopsis. Subsequently, at the 4-month corrected age follow-up appointment, confirmation of participation in the research is sought. Upon obtaining affirmative consent, informed consent forms are appropriately executed.

Parents have the right to withdraw their infant from the study at any time, for any reason, and without providing an explanation. Additionally, if the family discontinues health care at the institution where the study is being conducted, the infant will automatically be withdrawn from the study. The infant may also be withdrawn by the research team in the event of any unexpected or adverse reactions, or if they are unable to cooperate during the assessment procedures.

### Assessment protocol presentation

The study will be conducted in the infants’ first year, more specifically at 4-, 6-, 9- and 12-months age. The assessment includes clinical variables and sociodemographic data collection, the application of Bayley Scale of Infant and Toddler (to evaluate in a quantitative manner the motor and global development), the PAI-CY (to identify any potential participation limitation due to visual issues), the Electrophysiological measurements (VEP and EDA) and the Ophthalmological Screening Assessment.

**Fig. 2:**
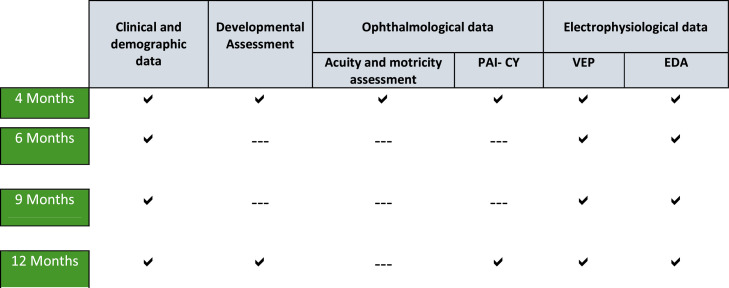
Data collection workflow: applied in both full-term and preterm infant groups.

In the second (6 months) and third (9 months) assessment, the data collection includes exclusively the electrophysiological measurements (VEP, EDA) and registration of clinical data collected by the Neonatologist / Physician, in the routine medical appointment (Fig. 2**.**). This option is taken to not overload the family and the infant and to minimize the possible test-retest effect related with development scale application. VEP and EDA are collected at all time points because they provide objective data based on infants’ physiological responses and are, according to the literature, among the least explored parameters in healthy preterm infants.

### Assessment sessions

**Fig. 3:**
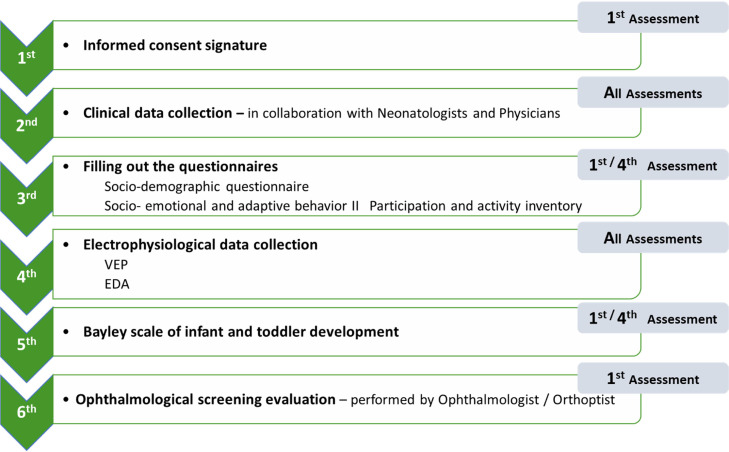
Typical assessment session under study with reference to assessment moments in each procedure.

As previously indicated, the assessment procedures vary slightly across sessions. The global workflow of data collection follows the structure presented in Fig. 2. The first session is presented as the reference model, with any necessary adaptations in subsequent sessions detailed accordingly (see Fig. 3**.).**

In the first session, the informed consent is applied and following that, the clinical data collection is performed. The data collected is slightly different in each session. In the first one the Neonatologist (in MAC) or the Physician (in Baixa Family Health Unit) share the data about pregnancy, medical history (maternal age, maternal diabetes, pre-eclampsia, gemellarity, among others) infant clinical parameters at birth (e.g.: spontaneous or medical assisted pregnancy, type of labour delivery, sex, race, birth weight, APGAR index, reanimation procedures, prenatal corticosteroids, invasive ventilation, ROP, leukomalacia, intraventricular haemorrhage and the actual somatometric parameters (weight, length, cephalic perimeter). In the next assessments, only the somatometric parameters are collected.

Following data collection regarding medical history, the questionnaires (Socio-demographic questionnaire, Socio-emotional and adaptative behaviour and the PAI-CY) are filled out in the first (4 months) and fourth (12 months) assessment.

Electrophysiological data collection constitutes one of the core procedures of this study. Both VEP and EDA are recorded at all assessment time points.

Environmental conditions and equipment placing on the infant for electrophysiological data collection

All assessments are conducted in quiet, semi-dark, and individualized rooms specifically designated for infant evaluations. Given that environmental factors may subtly influence electrophysiological recordings, a dedicated space with controlled and consistent conditions was identified within each participating healthcare institution. These rooms were reserved exclusively for study-related assessments throughout the data collection period to minimize variability and reduce the risk of environmental bias associated with conducting evaluations in heterogeneous settings.

**Fig. 4:**
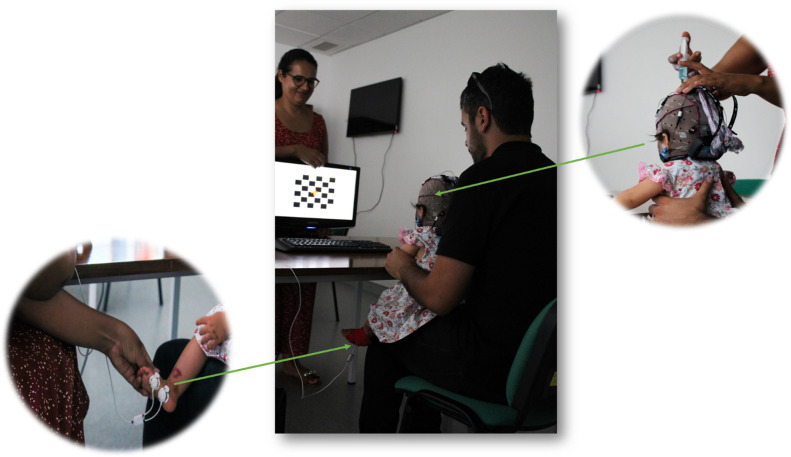
Participant seated on the parent's lap in front of the pattern-reversal checkerboard stimulus. A zoomed-in view is provided to illustrate electrode preparation for VEP recording and electrode placement for EDA data collection. (Image obtained with prior written consent from the parents).

The infant is seated in parents’ lap and positioned in front of a 19″ monitor 70 cm away, as represented in Fig. 4.

All the equipment runs on a Dell Computer (Intel ® Core (TN) 2DUO, 4,00 GB) that projects the image in a LG flatron W1934s 19′’monitor.

For EDA collection, the Biosignalsplux® is used with opensignals® software to record the signal [40]. Following the literature recommendations, the electrodes for EDA collection are placed on the external border of the foot [41]. These are connected through the cable to Biosignalplux® and that is connected via bluetooth to opensignals® software [40].

Once the proper connection between the equipment and the EDA collection software is established, the g.Nautilus® device (serial number NB-2017.08.77) is used with g.Recorder® software version 5.16.00 to record the VEP. The gNautilus® headset is fixed onto the cap and connected to the base station using a WiFi connection. The base station then sends the information to the g.recorder® using USB cables [42]. The cap is prepared with Oz, PO7, PO8 and Cz being the active electrodes, complemented with ground (positioned in Fz) and ear lobe electrode as the reference.

### Visual stimuli and its presentation

The visual stimuli consist of a black and white checkerboard image composed of 6 × 6 black and white squares, with a red cross positioned at the centre. The image alternates every second and is presented for a duration of 120 s. In supplemental material a short video from the visual stimuli presentation during an infant assessment can be found. The stimuli are generated and displayed using E-Prime® software [43]. This software was deliberately selected for its ability to present visual stimuli and simultaneously transmit event triggers via the parallel port to g.Recorder® and via socket to OpenSignals® [43]. These functionalities are essential to ensure temporal synchronization between stimulus presentation and signal acquisition, as well as alignment across different data streams.

This choice of software was deliberate due to its capability to present stimuli and send triggers via both the parallel port to g.Recorder® and via socket to OpenSignals® [43]. These features are significant as they enable synchronization between the stimuli presentation and the recorded data, as well as synchronization between different types of data.

### Data recorder

The VEP and EDA data are recorded in g.Recorder® and Opensignals® software, during the 120 s that checkboard stimuli is reversed. At the end of the presentation both software programs are turned off and automatically generate a Hierarchical Data Format (HDF5) for EEG signals and two files (text and H5) for EDA data.

### Developmental assessment

The Bayley Scale of Infant and Toddler Development is applied by the 1st author in the first and fourth assessment moments (4- and 12- months age), as presented in Fig.2. and Fig.3. The stimuli are presented following the procedures recommended in the administration manual [44]. In the study group, the correct age is used to decide which task should be used to begin the assessment. Then, the protocol for implementing the scale is followed.

### Ophthalmological screening evaluation

The retina is routinely assessed in preterm infants by a pediatric ophthalmologist in the days following delivery. Information regarding the presence of retinopathy of prematurity (ROP) is documented in the clinical records and subsequently retrieved for analysis in the present study.

The ophthalmological screening evaluation conducted specifically as part of the protocol is performed at four months of age by an orthoptist, with the aim of assessing visual acuity and ocular motricity and ensuring that these factors do not influence future VEP and PAI-CY results. Given the early age of the infants, discriminative visual acuity is measured in cycles/cm using the preferential looking method by applying Teller Cards as recommended in the literature [45]. The assessment is complemented through visual performance observation.

### Outcome measure description and contribution to preterm follow up

Next, the outcomes extracted from the different instruments and their contribution for reaching the prior established goals will be explained. The data will also be shared in open science repository Zenodo with the https://doi.org/10.5281/zenodo.10949757.

### Clinical and socio-demographic variable collected

The full list of Socio-Demographic and clinical variables collected during the research can be consulted in the supplemental material (Table 1). This data will be analyzed in order to characterize the sample and to obtain information to the study’s third aim.

**Table 1:** Main clinical and socio-demographic variables.

Variable	Full-term	Preterm	Variable (at 12 months)	Full-term	Preterm
Gender	Male	Female	Hours of sleep (per day)	6 h	12 hours
APGAR index (1st minute)	9	7	Daycare	Yes	No
APGAR index (5th minute)	10	9	Rehabilitation treatment	No	No
Labor delivery type	Eutocic	Eutocic	Medicines 4th assessment	No	No
Family	Mother	Father & Mother			
Siblings	No	No			
Mother age	19	33			
Mother’s academic degree	Not applicable	Bachelor			
Mother’s profession	Student	Plastic artist			
Medicines 1st assessment	No	Iron, Vitamin D, Multivitamins			

Somatometric measurements are recorded at all time points due to their importance for overall infant health, as they serve as key indicators of general health status. Table 2 presents the measurements taken from each infant at the four assessment time points.

### Outcomes measure extracted from motor development

The Bayley Scale of Infant Development (3^rd^ edition) was employed for assessment. Given the absence of a normative base specific to the Portuguese population, the approach involved comparing the raw data obtained from the two groups under study. Specifically, comparisons were made between scores obtained for motor domain at 4 and 12 months of age.

### Outcomes measure extracted from PAI-CY

In relation to the PAI-CY, the decision was made to extract results pertaining to each component: activity and participation, parent component, and sensory function component. The results will be compared between the groups and within the groups at first (4 months) and fourth (12 months) assessment moments, documenting the infants’ evolution.

### Electrophysiological data analysis and respective outcomes

The electrophysiological data is processed using two distinct scripts implemented in Matlab® 2024b and Ledalab® (specifically for EDA analysis). Although the scripts operate independently, they are integrated to facilitate the analysis. Their shared goal is the quantification and characterization of changes in the electrophysiological signal elicited by the stimuli.

The script for processing the VEP signal involves the following main steps:

1. Applying a 4th order Butterworth bandpass filter with a cutoff frequency of 2 Hz to 30 Hz, as suggested by the literature [[46], [47], [48]];
2. Selecting, throughout visual inspection, a temporal period with fewer artifacts, to calculate the standard deviation of the signal (Fig. 5**A**);

**Fig. 5A:**
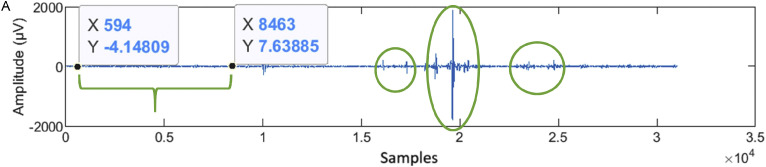
Workflow for data processing exemplified in a preterm infant 1st assessment. **A.** A temporal period with fewer artifacts that will be selected to calculate the standard deviation is showed in the green keystone. The main artifacts, to be removed for signal processing, are indicated with the green ellipses.
3. Using the standard deviation value, which will be multiplied empirically by 1.5, to define the cutoff threshold for excluding artifacts;
4. Locating the triggers within the file;
5. Using recorded triggers to divide the signal in epochs of 700 ms each (100 ms before each stimulus, and 600 ms after);
6. Identifying the epochs with artifacts;
7. Averaging the epochs free from artifacts, to obtain the evoked potentials waveforms;
8. Applying a Butterworth low-pass filter with a cutoff frequency of 10 Hz.

A series of methodological strategies were implemented to address signal artifacts during electrophysiological data processing. Initially, major artifacts were identified through visual inspection, as illustrated in Fig. 5**A** (green ellipse). Subsequently, a segment of the signal exhibiting stable baseline characteristics was selected, and its standard deviation was calculated. A threshold for acceptable signal variation was then defined as 1.5 times this standard deviation, allowing for adaptive filtering of the full signal.

The signal was segmented into epochs, each comprising 20 stimuli, within a total stimulation period of 120 s (Fig. 5. B). From the six resulting epochs, those with minimal artifact presence and a clearly discernible P100 component were selected for further analysis. These selected epochs were used to compute the final P100 amplitude and latency values (Fig. 5. B**.**). The P100 complex was then identified and confirmed in the averaged visual evoked potential waveform, as shown in Fig. 5. C.

**Fig. 5B:**
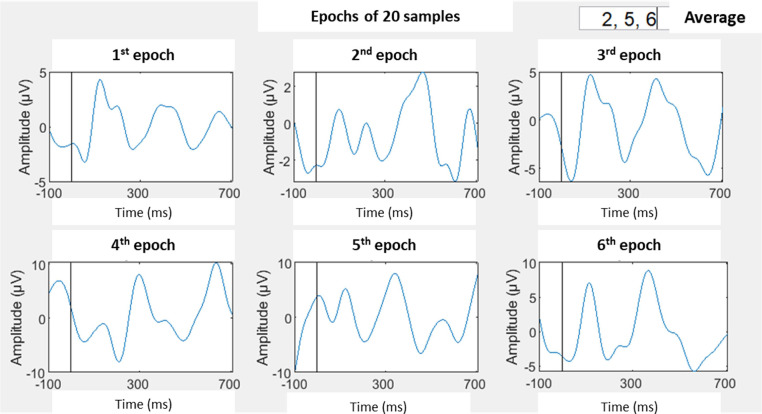
Six epochs resulting from artifacts removal and standard deviation application, followed by epochs selection.

**Fig. 5C:**
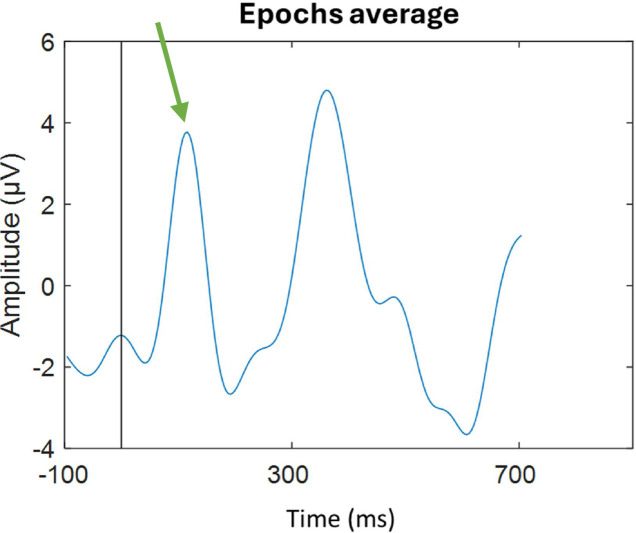
Signal obtained from Oz channel in the 1st assessment, with P100 pointed out by the green arrow.

It is important to note that the accurate identification of the P100 complex, as well as effective artifact management, required the application of the procedures outlined above to ensure data reliability and interpretability.

Finally, the N75, P100, and N135 components are identified, and their amplitude and latency are measured at the Oz electrode, considering that position corresponds to the primary visual cortex and are the most frequently characterized in VEP [22]. Given the developmental characteristics of brain maturation in infants as young as those assessed in the present protocol, some deflections may not be detectable at the Oz (mostly in young infants). In such cases, the corresponding parameters will be examined in adjacent electrodes, PO8 and PO7.

For the processing of the EDA signal, the Ledalab® software is employed. Initially, MATLAB® 2024b is used to downsample the data from 500 Hz to 100 Hz, considering the slow nature of EDA signals [49]. Subsequently, the raw data is converted to international units (microsiemens - µS). Following these data adjustments, a 50-point moving average filter is applied to smoothen the signal.

**Fig. 6:**
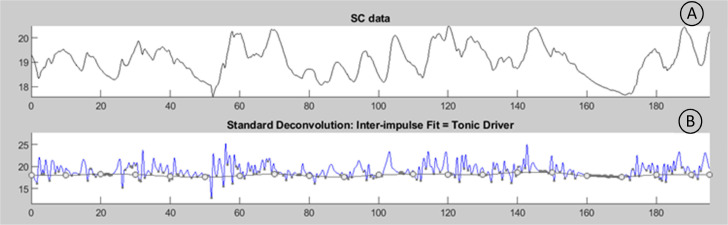
EDA signal: A – original skin conductance data; B – Standard deconvolution of skin conductance data: the tonic driver is observed.

Both trigger and EDA data files are imported into Ledalab® to conduct the continuous measure of phasic activity which enables the separation of the tonic and phasic components of the signal [39] and the EDA parameters are extracted. In Fig. 6. the first steps used in this approach are presented.

The signal is then analysed, and a specific skin conductance response (SCR) window of 1 to 4 s with a 0.01 µS SCR amplitude is utilized. Inspired by the literature [41,50] it will register the number of skin conductance responses, latency (in seconds), amplitude (in µS), phasic driver (in µS), area (in (µS)^2^) and average of tonic component (in µS). Those parameters will be compared between groups and within each group to quantify the evolution.

### Statistical power and sample size calculation

The sample size was calculated using G*Power version 3.1, assuming a repeated-measures ANOVA with a within-between interaction (2 groups × 4 time points). The following parameters were defined: statistical power of 80 % (1–β = 0.80), significance level α = 0.05, and an expected large effect size (*f* = 0.35), as defined by Cohen [51].

As no previous studies were identified using a longitudinal protocol integrating electrophysiological, developmental, and clinical data in preterm and full-term infants, it was not possible to base the effect size on empirical evidence. Therefore, the decision to use a large effect size was grounded in theoretical expectations of meaningful developmental differences between the two groups, particularly regarding neurophysiological maturation throughout the first year of life.

Based on these assumptions, the minimum estimated sample size was 44 infants, with 22 participants in each group.

### Plan for statistical analysis

To evaluate developmental changes over time and between groups, a mixed-design repeated measures ANOVA will be performed, with time (4-, 6-, 9-, and 12-months) as the within-subjects factor and *group* (preterm vs. full-term) as the between-subjects factor. The assumptions of normality, sphericity (tested via Mauchly’s test), and homogeneity of variances (Levene’s test) will be examined. If the assumption of sphericity is violated, the Greenhouse–Geisser correction will be applied. Post hoc pairwise comparisons will be adjusted using the Bonferroni correction to control for multiple testing.

In addition, exploratory multiple linear regression models will be used to investigate the influence of clinical (e.g., gestational age, APGAR score, birth weight) and socio-demographic variables (e.g., mother age, parental education) on developmental outcomes. These outcomes include changes in Bayley motor scores, VEP latency/amplitude, and EDA parameters, calculated as the difference between the first and fourth assessment time points. Model assumptions will be verified, and multicollinearity will be assessed using the variance inflation factor. All analyses will be performed using SPSS version 30, with a two-sided significance level set at *p* < 0.05.

### Pilot application preliminary results

In order to illustrate the data obtained from the application of the present protocol, two cases will be presented as well as the respective data analysis. One case was selected from the control group (full-term infants) and the other case from the study group (preterm infants).

Clinical and socio-demographic variables were collected during infant medical appointments and through questionnaires administered at various stages, as previously outlined in assessment protocol presentation.

For the general characterization of the participants, it is important to clarify that the full-term infant was born at 40 weeks and 5 days of gestational age, with an APGAR score of 9 at the 1st minute and 10 at the 5th minute, as shown in Table 1, which summarizes the clinical and socio-demographic variables.

In contrast, the preterm infant was born at 29 weeks and 4 days of gestational age, with an APGAR score of 7 at the 1st minute and 9 at the 5th minute. Additional clinical, socio-demographic data can be found in Table 1. These variables are introduced to exemplify the protocol's application and are not discussed in detail. However, further analysis is planned to explore these variables and their potential correlation with visual and motor development, in line with the third aim previously described.

## Electrophysiological data

**Fig. 7:**
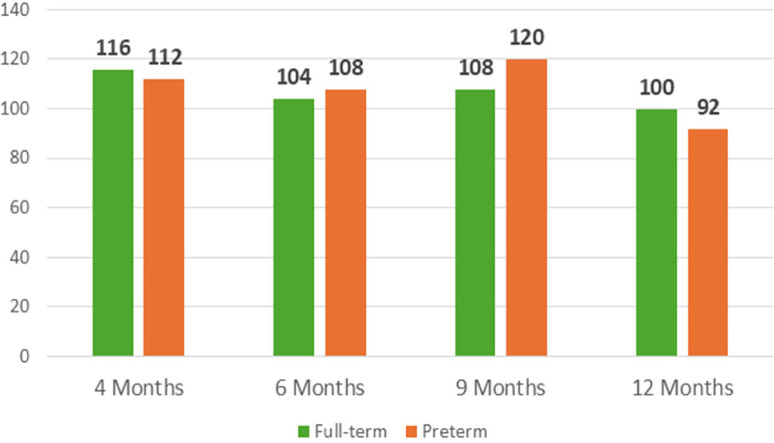
P100 Oz latency (in ms) across development in full-term and in preterm infants.

**Fig. 8:**
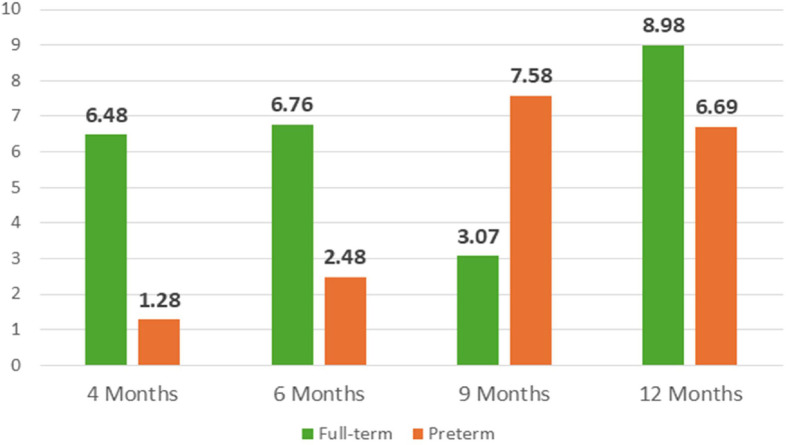
P100 Oz amplitude (in μV) across development in full-term and preterm infants.

Both VEP and EDA measures are taken in all the 4 assessment moments. Regarding the VEP waveform, the latency and amplitude of the three characteristic deflections—N75, P100 and N135— in Oz positioning were analyzed (supplemental material – Table 2). Fig. 7**.** and Fig. 8**.** illustrates the P100 component's latency and amplitude across four evaluation time points for both full-term infants and preterm.

**Table 2:** Somatometric measurements at birth and in each assessment moment.

		At birth	4 months	6 months	9 months	12 months
Weight (in kg)	Full-term	3.260	6.510	7.090	8.325	9.710
	Preterm	1.315	6.090	8.025	9.060	9.970
Height (in cm)	Full-term	50.1	64.0	66.5	72.0	76.0
	Preterm	40.0	57.5	64.7	72.2	77.5
Cephalic perimeter (in cm)	Full-term	36.0	42.6	44.0	45.0	47.0
	Preterm	27.9	41.2	45.0	46.0	48.0

**Fig. 9:**
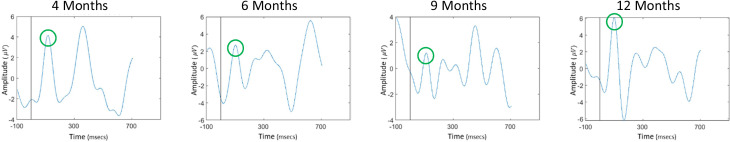
Longitudinal progression of VEPs in full-term infant across four assessment points during the first year of life. The P100 is indicated with a circle.

**Fig. 10:**
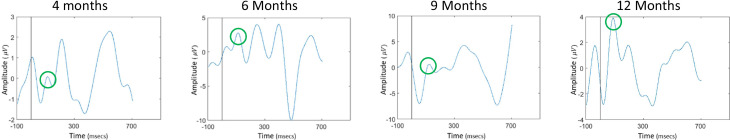
Longitudinal progression of VEPs in preterm infant across four assessment points during the first year of life. The P100 is indicated with a circle.

Furthermore, Fig. 9**.** and Fig. 10**.** depict the progression of VEP responses throughout the first year of life in both cohorts. Detailed numerical values corresponding to each deflection (N75, P100, N135), as presented in Fig.9**.** and Fig. 10**.,** are provided in Table 2 of the supplementary material.

If, for any reason (e.g., young infant age, atypical cortical development due to preterm birth), the identified complex cannot be detected, it is planned to be investigated in other secondary visual positions (PO8 and/or PO7).

Preliminary analysis of the VEP data supports the applicability of the current protocol. However, the data from these two cases do not allow for the identification of definitive trends in VEP responses. Nonetheless, in both cases, the waveform observed at 12 months of age appears more defined compared to those from earlier assessments. Additionally, the N75, P100, and N135 components presented by the full-term infant are more clearly defined than those observed in the preterm infant at the same age.

In the present study, EDA was analyzed using a continuous approach, wherein the TAU1 and TAU2 parameters optimization was performed to enhance the correlation between the error parameter and the negativity index. In this analysis, error values ranged from 1.84 to 4.13, while the negativity index varied between 0.18 and 0.55. In the present study, the EDA analysis is performed using a continuous analysis optimization, the values of TAU1 and TAU2 are adjusted with a view to achieving a superior correlation between error parameter and negativity index. In the present analysis, the error values are situated within the range of 1.84 to 4.13, while the negativity index is situated between 0.18 and 0.55.

**Fig. 11:**
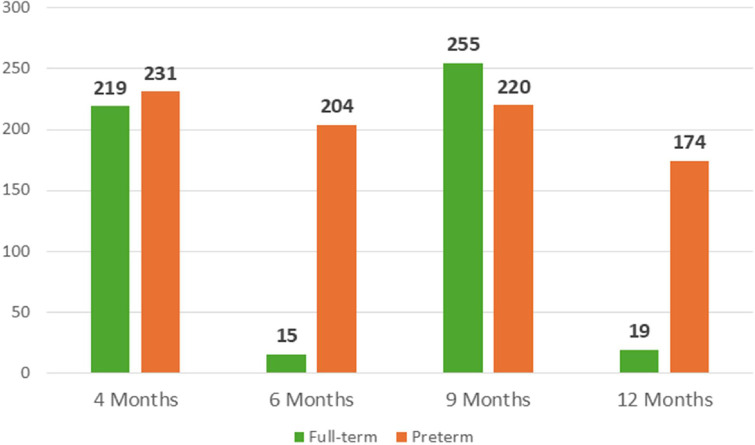
nSCR across development in full-term and preterm infants.

The application of the personalized parametrization yielded the results pertaining to the number of skin conductance responses, latency, amplitude and tonic component, as illustrated in Fig. 11**.** to Fig. 14**.** In order to provide further context and support of the findings presented in the main body of the text, the supplemental material should be consulted. Within the supplementary material, specifically in Table 3, additional parameters that pertain to the analysis of EDA can be found.

**Table 3:** Results from Bayley Scale of Infant and Toddler development (at 4 and 12 months).

	4 Months		12 Months	
	Full-term	Preterm	Full-term	Preterm
Fine motricity	9	5	29	29
Gross motricity	12	12	45	38

Fig. 12, Fig. 13

**Fig. 12:**
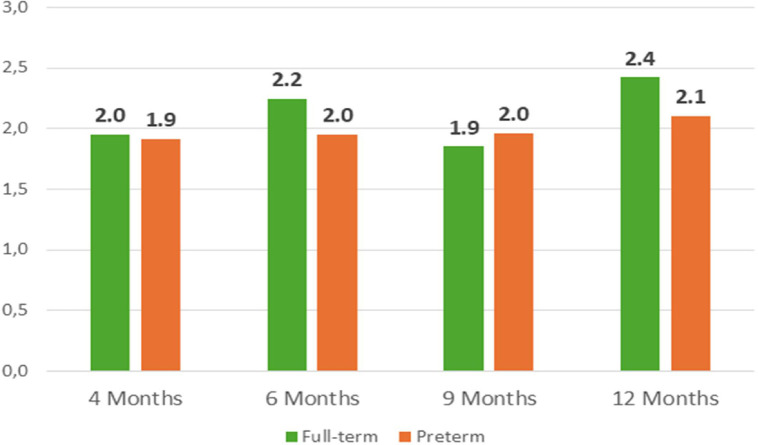
Latency (in s) across development in full-term and preterm infants.

**Fig. 13:**
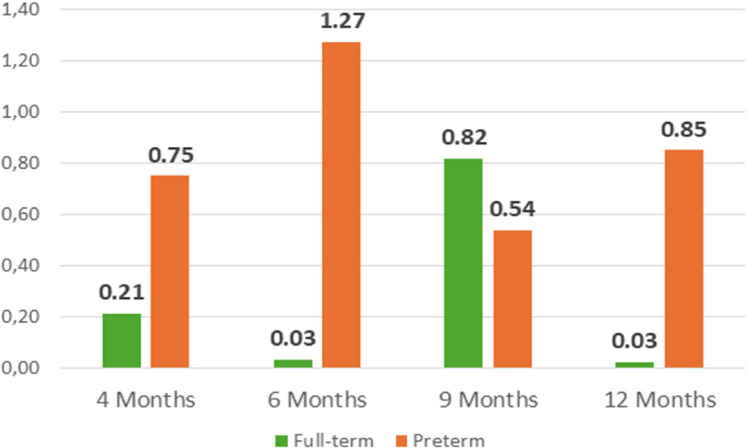
Amplitude (in μS) across development in full-term and in preterm infants.

**Fig. 14:**
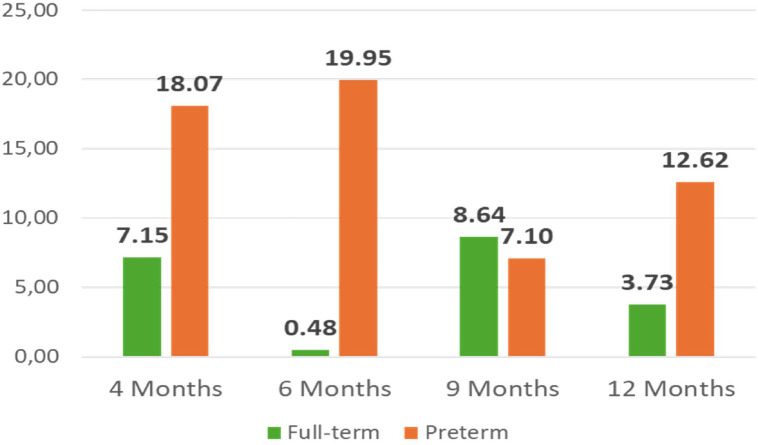
Tonic component (in μS) across development in full-term and in preterm infants.

To the best of our knowledge, the reverse pattern checkerboard is being used for the first time to elicit EDA activity. It is imperative that the results of this study are considered exploratory in nature, and that the data collected from all participants is integrated for the purpose of conducting a comprehensive analysis, exploring the hypothetical relation with motor development. However, the present pilot application demonstrates the executability of the procedures with the sample.

Regarding motor development, the Bayley Scales of Infant and Toddler Development were applied, and the results are presented in Table 3. In Table 4 the results obtained from the PAI-CY at 4 and 12 months of age are included. For both assessments, it is important to consider not only the total scores but also, and more importantly, the developmental progression of each infant.

**Table 4:** Results from PAI - CY (at 4 and 12 months).

	4 Months		12 Months	
	Full-term	Preterm	Full-term	Preterm
Activity and participation	47	18	76	80
Parent component	9	5	12	9
Sensory functioning component	29	18	31	28

It is important to emphasize that both the full-term and preterm participants demonstrated typical visual acuity and adequate ocular motility for their age, as documented by ophthalmological evaluation at 4 months of age.

The motor scores obtained using the Bayley Scales indicate a trend toward convergence between full-term and preterm infants at 12 months of age, particularly in the fine motor subscale. Results from the PAI-CY similarly reflect a close alignment of scores, especially in the domains of activity and participation, as well as sensory functioning.

In summary, the protocol development demonstrates that employing a co-creation methodology facilitates the optimization of experimental procedures, leading to a better alignment between the protocol and the characteristics of the sample and research context. Additionally, the preliminary information presented summarizes data collected during the protocol pilot implementation involving two cases: a full-term and a preterm infant. The findings support the feasibility of the protocol’s implementation in a pilot context.

### Limitations

For the assessment protocol to be effectively implemented, it must be conducted under standardized environmental conditions, as described in the subsection 'Environmental conditions and equipment placement for electrophysiological data collection'. Additionally, infants should be in a calm-alert behavioral state and recently fed to ensure optimal cooperation and optimize signal quality.

## CRediT author statement

**Ana Isabel Ferreira:** Conceptualization, Data curation, Formal analysis, Investigation, Methodology, Writing - original draft. **Alice Nunes:** Data Curation, Writing - original draft preparation. **Cláudia Ribeiro:** Data curation, Writing - original draft preparation. **Teresa Tomé:** Conceptualization, Methodology, Writing - original draft. **Cláudia Quaresma:** Conceptualization, Funding acquisition, Supervision, Writing - reviewing & editing. **Carla Quintão:** Conceptualization, Funding acquisition, Supervision, Writing - reviewing & editing;

## Related research article

“None”.

## For a published article

“None”.

## Declaration of competing interest

The authors declare that they have no known competing financial interests or personal relationships that could have appeared to influence the work reported in this paper.

## Data Availability

Data will be made available on request.

## Appendix Supplementary materials

Image, video 1

Supplementary material *and/or* additional information [OPTIONAL] Image, application 2
