# Supplementary material for: Visual and motor skills development in preterm and full-term infants: A protocol for longitudinal data collection
Source: MethodsX. 2025 Jul 30;15:103546. doi: 10.1016/j.mex.2025.103546 (PMC12341574; doi:10.1016/j.mex.2025.103546)
Supplement: Supplementary file 2 — Supplementary material and/or additional information [OPTIONAL] [file mmc2.docx]

**Supplemental Materials**

**Article:**

Visual and Motor Skills Development in Preterm and Full-term Infants: A Protocol for Longitudinal Data Collection

**Table 1**

Clinical and socio-demographic variables collected in both groups at different assessment points

|  |  |  | **Full-term** | **Preterm** |
| --- | --- | --- | --- | --- |
| **At birth (collected retrospectively)** | **Infant** | Gender | ✔ | ✔ |
|  |  | Gestational age | ✔ | ✔ |
|  |  | Birth weight | ✔ | ✔ |
|  |  | Birth length | ✔ | ✔ |
|  |  | Cephalic perimeter at birth | ✔ | ✔ |
|  |  | APGAR index 1^st^ minute | ✔ | ✔ |
|  |  | APGAR index 5^th^ minute | ✔ | ✔ |
|  |  | APGAR index 10^th^ minute | **---** | ✔ |
|  |  | Twinning | ✔ | ✔ |
|  |  | Monicorionic / bichorionic | ✔ | ✔ |
|  |  | Type of labor | ✔ | ✔ |
|  |  | Resuscitation | **---** | ✔ |
|  |  | Antenatal corticosteroids | **---** | ✔ |
|  |  | Neuroprotection | **---** | ✔ |
|  |  | Ventilation number of days | **---** | ✔ |
|  |  | Number of days hospitalized | **---** | ✔ |
|  |  | Sepsis | **---** | ✔ |
|  |  | Retinopathy of prematurity - right eye | **---** | ✔ |
|  |  | Retinopathy of prematurity - left eye | **---** | ✔ |
|  |  | Intraventricular haemorrhage - right hemisphere | **---** | ✔ |
|  |  | Intraventricular haemorrhage - left hemisphere | **---** | ✔ |
|  |  | Leukomalacia | **---** | ✔ |
|  |  | Transfontanelar ultrasound | **---** | ✔ |
|  | **Mother** | Diabetes type 1 | ✔ | ✔ |
|  |  | Gestational diabetes | ✔ | ✔ |
|  |  | Pre-eclampsia | **---** | ✔ |
|  |  | Placental abruption | **---** | ✔ |
|  |  | Chorioamnionitis | **---** | ✔ |

| **4 Months** |  | **Full-term** | **Preterm** |
| --- | --- | --- | --- |
|  | Infant weight | ✔ | ✔ |
|  | Infant length | ✔ | ✔ |
|  | Infant cephalic perimeter | ✔ | ✔ |
|  | Supplementation in first assessment | ✔ | ✔ |
|  | Medicines in first assessment | ✔ | ✔ |
|  | Nuclear family | ✔ | ✔ |
|  | Siblings | ✔ | ✔ |
|  | Age of siblings | ✔ | ✔ |
|  | Mother age | ✔ | ✔ |
|  | Father age | ✔ | ✔ |
|  | Mother nationality | ✔ | ✔ |
|  | Mother academic degree | ✔ | ✔ |
|  | Father academic degree | ✔ | ✔ |
|  | Mother profession | ✔ | ✔ |
|  | Father profession | ✔ | ✔ |
| **6 and 9 Months** | Infant weight | ✔ | ✔ |
|  | Infant length | ✔ | ✔ |
|  | Infant cephalic perimeter | ✔ | ✔ |
| **12 Months** | Infant weight | ✔ | ✔ |
|  | Infant length | ✔ | ✔ |
|  | Infant cephalic perimeter | ✔ | ✔ |
|  | Hours of sleep (per day) | ✔ | ✔ |
|  | Daycare | ✔ | ✔ |
|  | Age of daycare begining | ✔ | ✔ |
|  | Therapies (in past and currently) | ✔ | ✔ |
|  | Therapies (if does or have done, in which age and reason) | ✔ | ✔ |
|  | Medicines (in past and currently) | ✔ | ✔ |

**Table 1 (cont.)**

**Table 2**

VEP result from Oz channel (for full-term and preterm case)

|  |  | **N75** | | **P100** | | **N135** | |
| --- | --- | --- | --- | --- | --- | --- | --- |
|  |  | **Latency**  (in ms) | **Amplitude**  (in $\mu V)$ | **Latency**  (in ms) | **Amplitude**  (in $\mu V)$ | **Latency**  (in ms) | **Amplitude**  (in $\mu V)$ |
| **4** Months | Full-term | 40 | -2.31 | 116 | 6.49 | 192 | -6.50 |
|  | Preterm | 64 | -1.20 | 112 | 1.29 | 148 | -0.48 |
| **6** Months | Full-term | 20 | -4.05 | 104 | 6.76 | 168 | -5.05 |
|  | Preterm | 56 | 0.27 | 108 | 2.48 | 164 | -4.31 |
| **9** Months | Full-term | 56 | -1.91 | 108 | 3.08 | 168 | -3.52 |
|  | Preterm | 52 | -6.97 | 120 | 7.59 | 164 | -1.55 |
| **12** Months | Full-term | 32 | -2.88 | 100 | 8.99 | 172 | -12.22 |
|  | Preterm | 20 | -2.81 | 92 | 6.69 | 148 | -3.29 |

**Table 3**

EDA analysis using continuous decomposition analysis (for full-term and preterm case)

|  |  | **nSCR** | **Latency** (s) | **Amplitude** (uS)- sum | **Phasic Driver** (uS) | **Area** (uS^2) | **Tonic component** (uS)-average |
| --- | --- | --- | --- | --- | --- | --- | --- |
| **4** Months | Full-term | 219 | 1.95 | 0.21 | 0.0039 | 1.16 | 7.15 |
|  | Preterm | 231 | 1.91 | 0.75 | 2.8166 | 3.06 | 18.07 |
| **6** Months | Full-term | 15 | 2.25 | 0.03 | 0.0002 | 0.05 | 0.48 |
|  | Preterm | 204 | 1.95 | 1.27 | 4.5787 | 4.94 | 19.95 |
| **9** Months | Full-term | 255 | 1.85 | 0.82 | 0.0083 | 2.48 | 8.64 |
|  | Preterm | 220 | 1.96 | 0.54 | 2.1111 | 2.28 | 7.10 |
| **12** Months | Full-term | 19 | 2.42 | 0.02 | 0.0001 | 0.04 | 3.73 |
|  | Preterm | 174 | 2.10 | 0.85 | 3.5032 | 4.19 | 12.62 |
